# Uptake of COVID-19 vaccinations amongst 3,433,483 children and young people across four nations in the UK

**DOI:** 10.23889/ijpds.v9i5.2736

**Published:** 2024-09-18

**Authors:** Sarah J Aldridge, Utkarsh Agrawal, Siobhán Murphy, Tristan Millington, Ashley Akbari, Fatima Almaghrabi, Sneha N Anand, Stuart Bedston, Rosalind Goudie, Rowena Griffiths, Mark Joy, Emily Lowthian, Simon de Lusignan, Lynsey Patterson, Chris Robertson, Igor Rudan, Declan T Bradley, Ronan Lyons, Aziz Sheikh, Rhiannon K Owen

**Affiliations:** 1 Population Data Science, Swansea University Medical School, Faculty of Medicine, Health, and Life Science, Swansea University; 2 Nuffield Department of Primary Care Health Sciences, University of Oxford; 3 Centre for Public Health, Queen’s University Belfast; 4 Usher Institute, University of Edinburgh; 5 Department of Education and Childhood Studies, School of Social Sciences, Swansea University; 6 Centre for Public Health, School of Medicine, Dentistry and Biomedical Sciences, Queen’s University Belfast, Belfast, UK; 7 Public Health Agency; 8 Department of Mathematics and Statistics, Strathclyde University; 9 Public Health Scotland; 10 Centre for Global Health, Usher Institute, the University of Edinburgh

## Background and Objectives

SARS-CoV-2 infection in children and young people (CYP) can lead to life threatening conditions including COVID-19, transmission to more vulnerable individuals, or even long COVID. Vaccination against COVID-19 reduces the chance of infection and transmission of the virus, but vaccine uptake in the UK has been shown to decrease with age.

## Approach

We undertook a multistate-model approach to estimate hazard ratios on national cohorts constructed from linked health and administrative data, adjusting for several demographic factors. The models were applied to 3,433,483 CYP aged 5-17 years between 4th August 2021 and 31st May 2022. The results were combined in a random effects meta-analysis.

## Results

Uptake of the first COVID-19 vaccine in CYP was lower compared to older age groups in the UK, and diminished further with subsequent doses (34%, 20% and 2% for 1st, 2nd, and booster dose respectively). Age of the CYP and vaccination status of the adults in the household were identified as important risk factors. For example, 5-11-year-olds were less likely to receive their first vaccine compared to 16-17-year-olds (adjusted Hazard Ratio [aHR]: 0.10 (95%CI: 0.06-0.19), and CYP in unvaccinated households were less likely to receive their first vaccine compared to partially vaccinated households (aHR: 0.19, 95%CI 0.13-0.29).

## Conclusions and Implications

Our work highlights the need for targeted strategies to increase COVID-19 vaccine acceptance and uptake among CYP, especially considering the influence of parental consent and household factors. Further research could help identify risk factors in household types to investigate more direct associations, which may be influenced by factors such as living with vulnerable people, familial structure, or deprivation.

